# GIDEON: a comprehensive Web-based resource for geographic medicine

**DOI:** 10.1186/1476-072X-4-10

**Published:** 2005-04-22

**Authors:** Stephen A Berger

**Affiliations:** 1 Department of Geographic Medicine, Tel Aviv Medical Center, 6 Weitzman Street, Tel Aviv 64239, Israel

## Abstract

GIDEON (Global Infectious Diseases and Epidemiology Network) is a web-based computer program designed for decision support and informatics in the field of Geographic Medicine. The first of four interactive modules generates a ranked differential diagnosis based on patient signs, symptoms, exposure history and country of disease acquisition. Additional options include syndromic disease surveillance capability and simulation of bioterrorism scenarios. The second module accesses detailed and current information regarding the status of 338 individual diseases in each of 220 countries. Over 50,000 disease images, maps and user-designed graphs may be downloaded for use in teaching and preparation of written materials. The third module is a comprehensive source on the use of 328 anti-infective drugs and vaccines, including a listing of over 9,500 international trade names. The fourth module can be used to characterize or identify any bacterium or yeast, based on laboratory phenotype. GIDEON is an up-to-date and comprehensive resource for Geographic Medicine.

## Introduction

As of 2005, the world is confronted by 338 generic infectious diseases, scattered in a complex fashion across over 220 countries and regions. Each new day confronts health care workers with unexpected outbreaks, epidemics and heretofore unknown pathogens. Over 2,000 named bacteria, viruses, fungi and parasites are known to cause human disease; and are confronted by 328 anti-infective agents and vaccines. Experts working in Health Geographics share an obvious and immediate need for comprehensive and timely data on the status of infection around the globe. A recent outline of GIDEON addressed uses for the Infectious Diseases clinician [1]. This review will focus on the Global Health aspect of the program.

In 1990, we initiated a project to design computer systems to follow all diseases, outbreaks, pathogens and drugs. The initial DOS-based program was written in Paradox for floppy disks, later evolving through a compact disk-based program in Windows. A commercial web-based program was eventually released under the name, GIDEON (Global Infectious Diseases and Epidemiology ON-line, Gideon Informatics, Inc, Los Angeles, California) at . The current version is available on CD (updated every three months) or web subscription (updated every week).

The program consists of four modules: Diagnosis, Epidemiology, Therapy and Microbiology. Program modules of peripheral interest in Health Geographics (Therapy and Microbiology) will be discussed only briefly.

## Diagnosis Module

**Figure 1:**
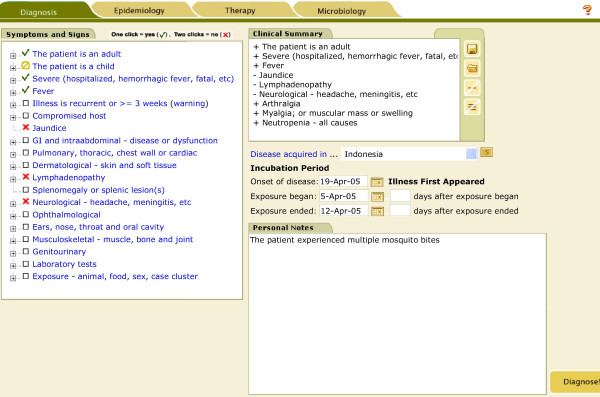
GIDEON Diagnosis module. Clinical data entered for a patient suffering from joint pain and fever following a trip to Indonesia.

**Figure 2:**
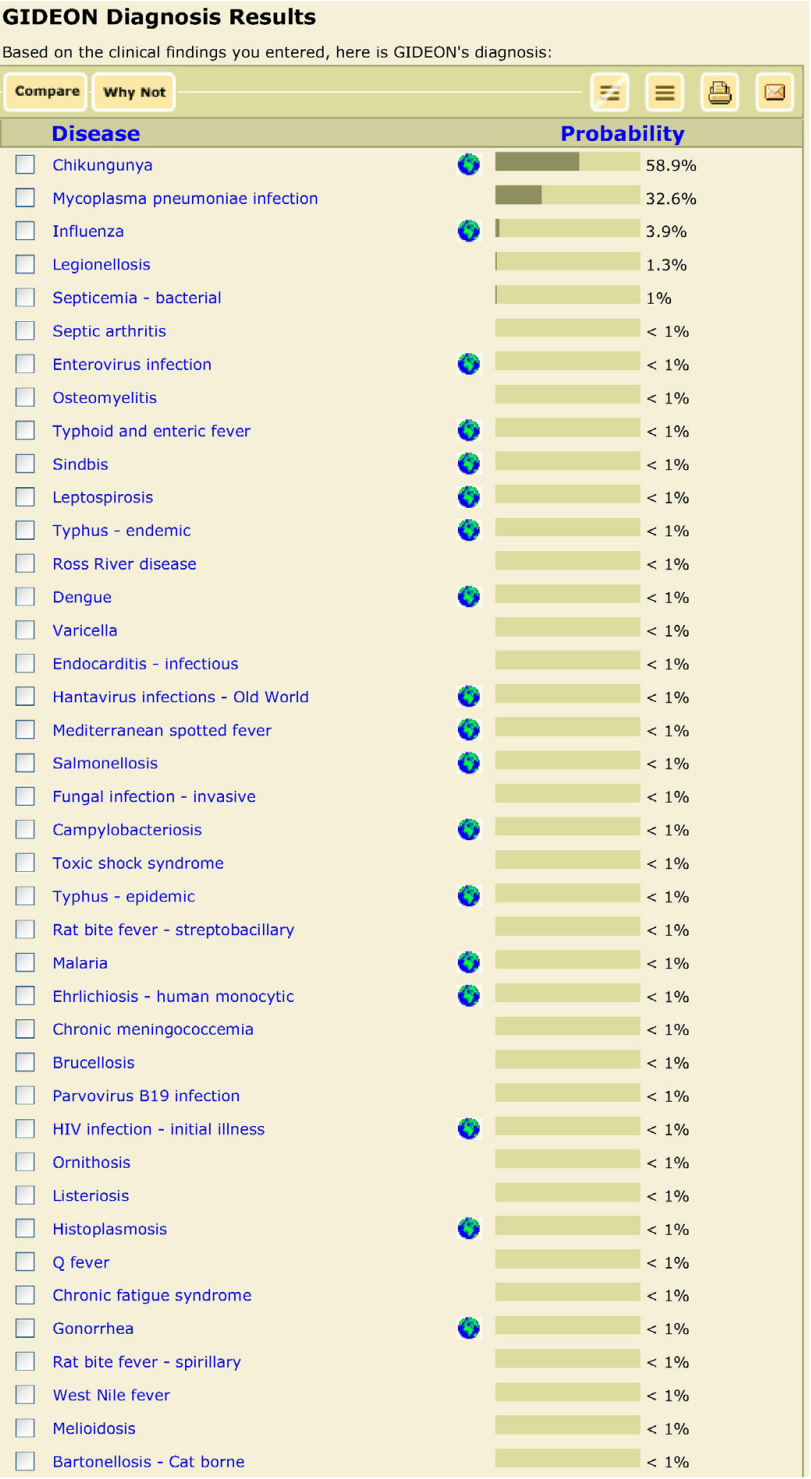
Differential diagnosis for case in Figure 1. "Why not" and 'Compare' options are available at upper left.

The Diagnosis module is designed to generate a ranked differential diagnosis based on signs, symptoms, laboratory tests, incubation period, nature of exposure and country of disease origin. Figure 1 depicts the data entry screen for a patient suffering from fever and joint pain following a trip to Indonesia. The lower 'Personal notes' box is used to record additional case data, and can be written in the user's own language. The differential diagnosis list for this case (figure 2) indicates that this patient may be suffering from Chikungunya. The appearance of many diseases on the list indicates that the user failed to enter all positive, and negative findings. For example, the fact that cough was absent would have reduced the likelihood of the second disease listed (Mycoplasma infection) and increased the statistical probability of Chikungunya.

**Figure 3:**
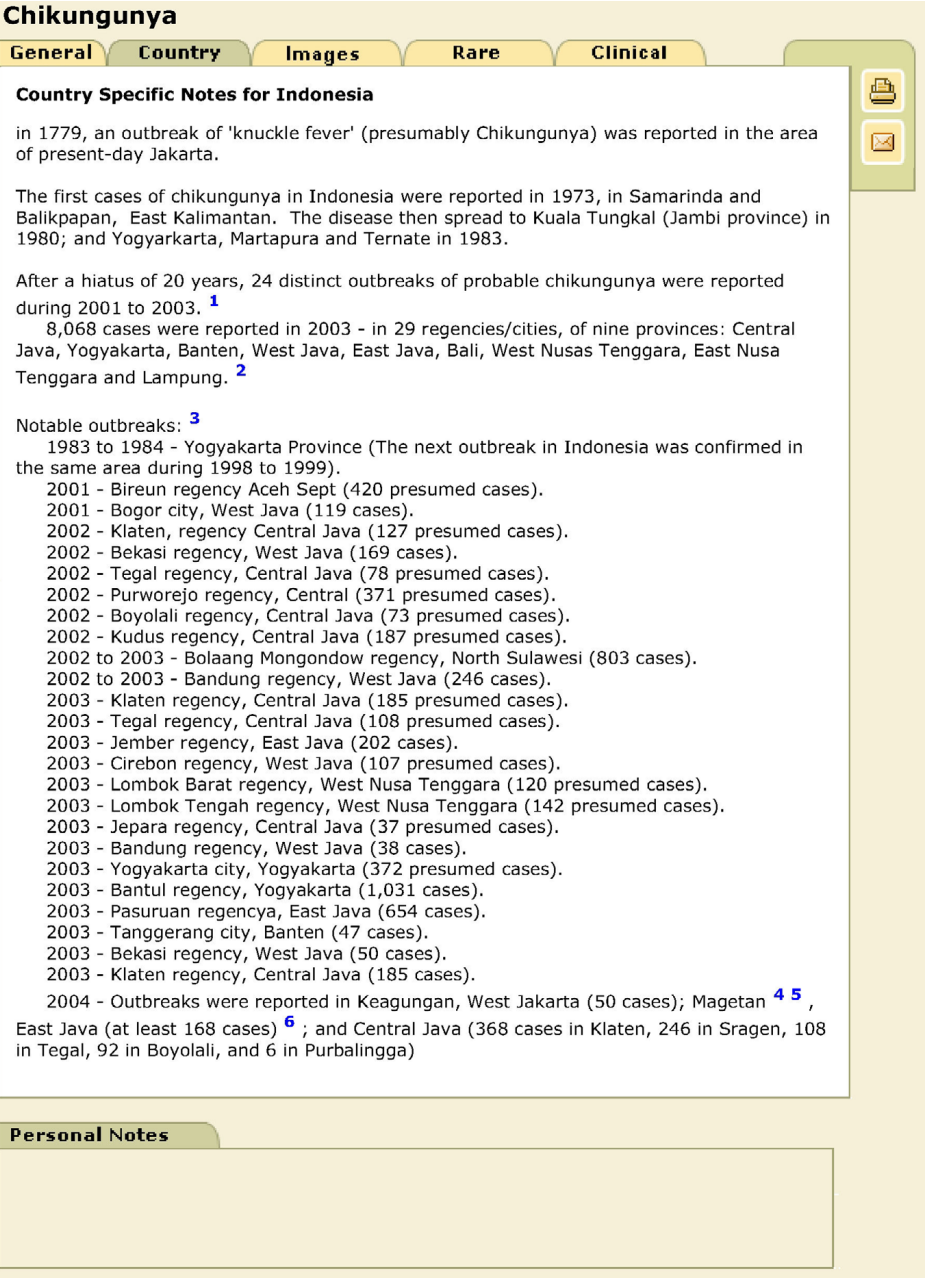
Epidemiological background on the status of Chikungunya in Indonesia. Additional options access the descriptive epidemiology ('General' tab), clinical features, clinical images, etc for this disease.



At this point, the user can generate a hard copy or e-mail report, access a table comparing the clinical features of the diseases listed, or examine the ranking or omission of specific diseases. If the user clicks on a specific disease name, clinical and epidemiological data on the disease in question are depicted (figure 3). The differential diagnosis list is generated by a Bayesian formula which compares the product of disease-incidence and symptom incidence, for all compatible infectious diseases. In the above example, a number of diseases known to occur in Indonesia were capable of producing fever, and joint pain. The statistical likelihood of Chikungunya in this case can be computed by a simple Bayesian formula, as follows:

C = Chikungunya, P = probability or incidence, S = observed symptoms

P-(C/S) = probability of Chikungunya, given these symptoms

D2, D3, Dn = other diseases compabible with this clinical scenario

Two spreadsheets in the GIDEON database respectively follow the incidence of all symptoms for every disease, and the incidence of all diseases for every country. When a clinical case is "entered" into GIDEON, the program identifies all compatible diseases and ranks their relative likelihoods as determined by the above formula, ie: P-(C/S) vs. P-(D2/S) vs. P-(D3/S) ... vs. P-(Dn/S).

A blinded study of 500 cases conducted by this author found that the correct diagnosis was listed in the differential list in 94.7% of cases, and was ranked first in 75% [2]. A second study of hospitalized patients in Boston found that the correct diagnosis was listed in only 69%, and was ranked first in 60% [3]. It is likely that inclusion in the differential diagnosis list may be more important than disease ranking in such systems [4].

**Figure 4:**
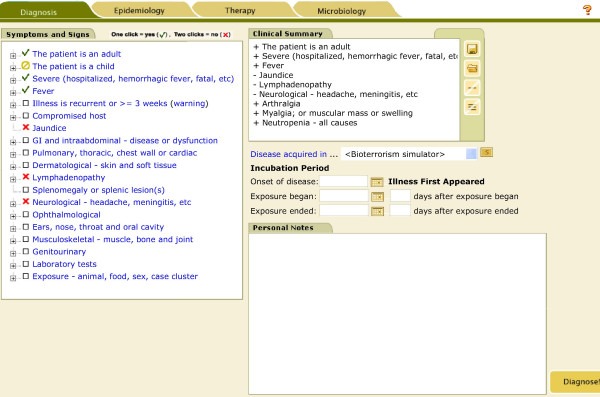
Data entry screen for a bioterrorism scenario.

A "Bioterrorism" option generates the differential diagnosis for diseases associated with suspected bioterror scenarios. In Figure 4, "<bioterrorism simulator>" has been substituted for Indonesia, given the above constellation of fever, joint pain, etc. The resulting differential diagnosis lists Ebola (42.9% probability), followed by Crimean-Congo hemorrhagic fever (12.6% probability). A similar "Worldwide" option can be used to explore all of the worlds diseases consistent with given clinical features, and access text on the global status for individual diseases.

In theory, data entry by users can be monitored at the server level for purposes of surveillance. For example, if one or more users in China were to enter cases of fatal pneumonia, a "red-flag" at any monitoring agency (i.e., the World Health Organization) could indicate the possible appearance of SARS – long before submission of specimens or reporting of the case to local authorities. Similarly, the appearance of multiple cases of "dysentery" by users in a given community could indicate a possible outbreak of shigellosis.

## Epidemiology module

**Figure 5:**
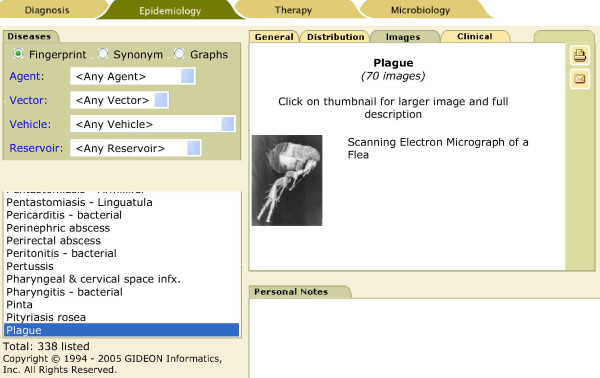
Epidemiology module, main screen. The 'images' tab has been pressed, to access thumbnail images of Plague. These can be maximized and copied to PowerPoint, etc. Note addition of 'Personal notes' by the user, at lower right.

The Epidemiology module presents detailed country-specific information on the status of each disease, both globally and within each relevant country. The current version contains over two million words in 12,000 notes. All data are derived from Health Ministry publications, peer-review journals, standard textbooks, WHO and CDC websites and data presented at conferences. The user may also access over 30,000 graphs which follow disease incidence, rates and other numerical data. The main Epidemiology screen is shown in Figure 5. Note that the user can append custom "personal notes" – in any national language or font- regarding the status of every disease in their own institution. Such notes would be accessible by all colleagues using GIDEON on the local network.

**Figure 6:**
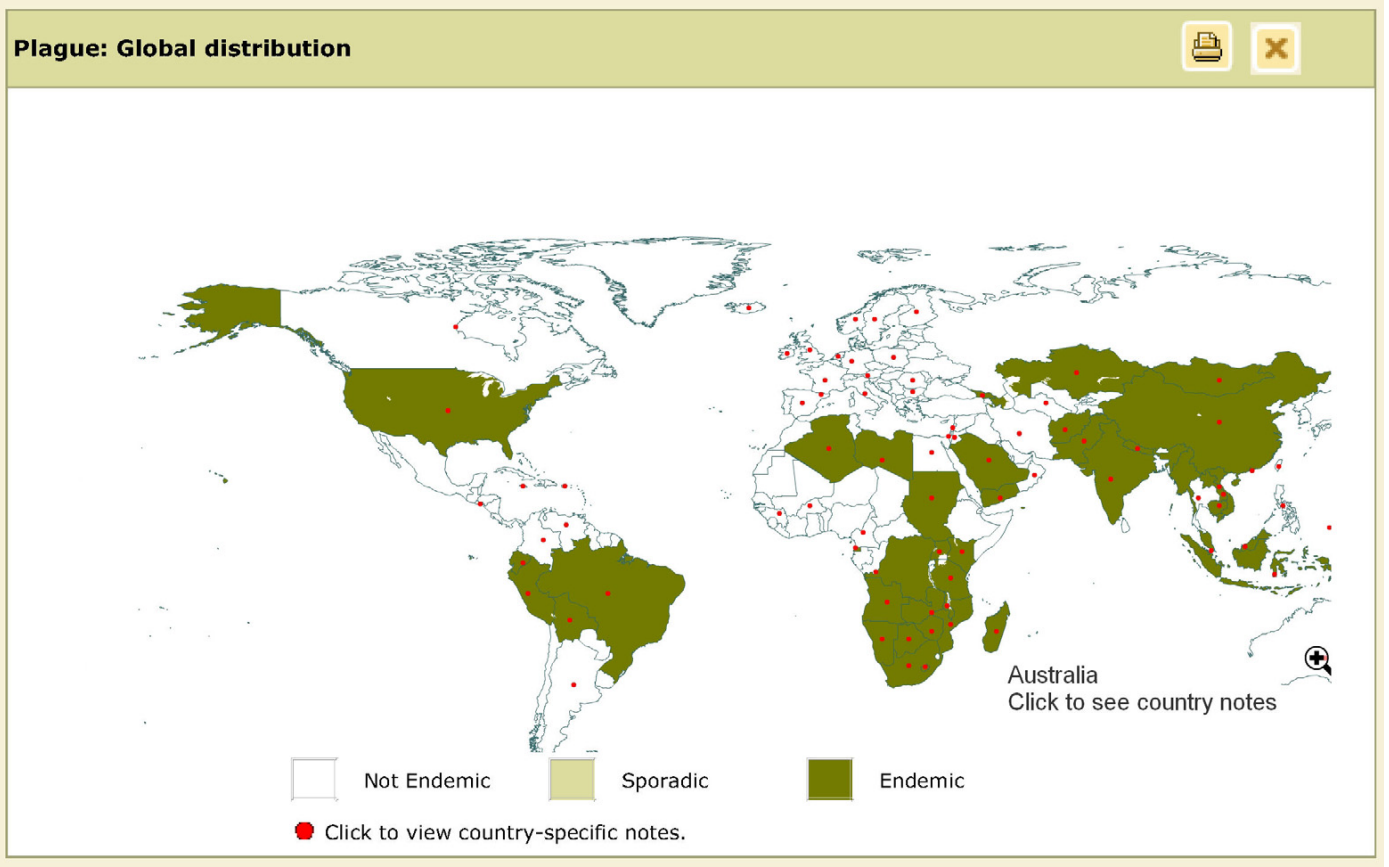
Epidemiology module. Map depicting the global distribution of plague. Specific map areas can be expanded, and all elements can be copied for reproduction as necessary. Country-specific notes regarding plague appear when corresponding red dots are clicked.

**Figure 7:**
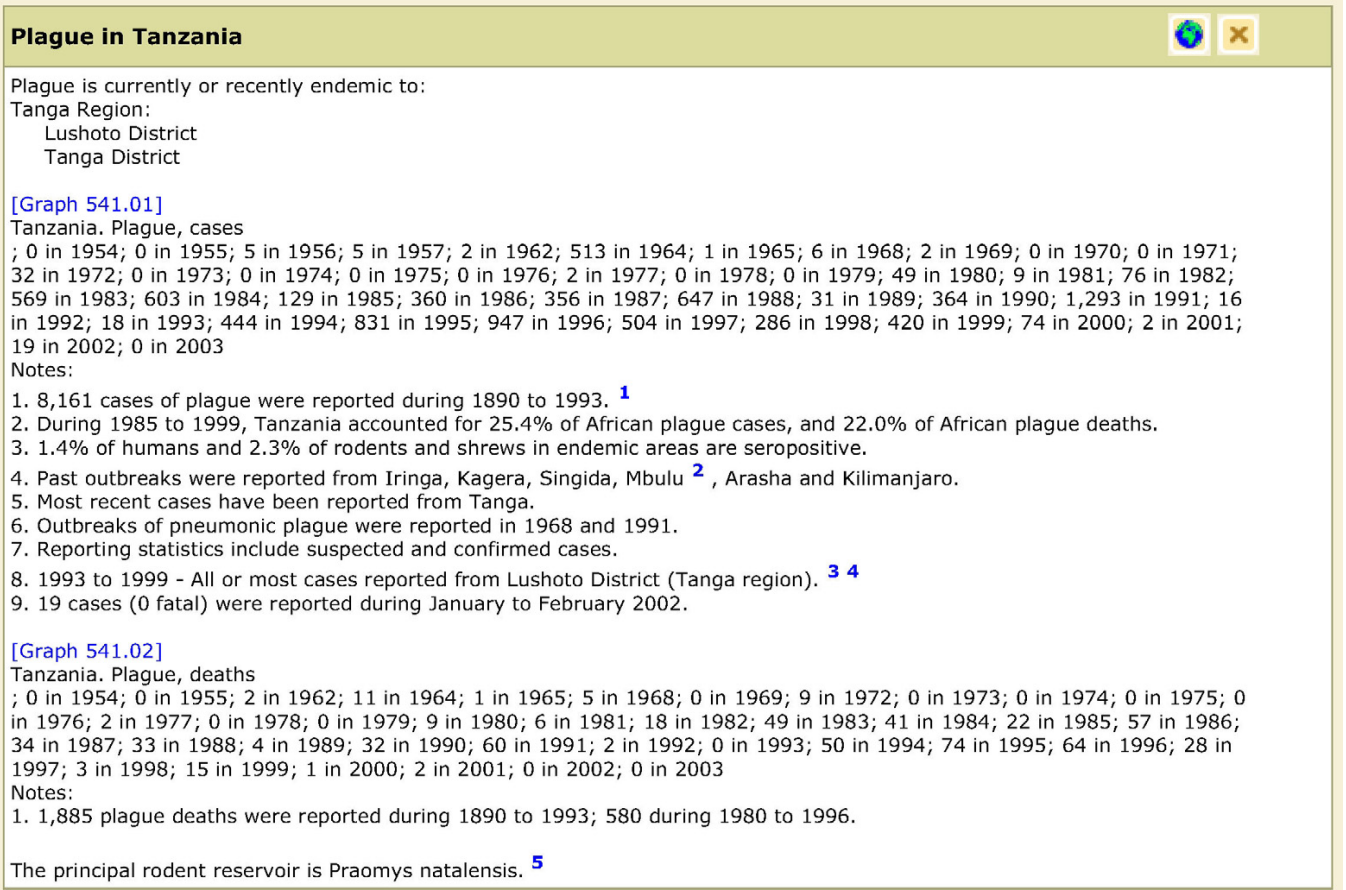
Plague in Tanzania. Clicking on relevant data sets will generate incidence and rates graphs. Note several numbered links to journal publications.

**Figure 8:**
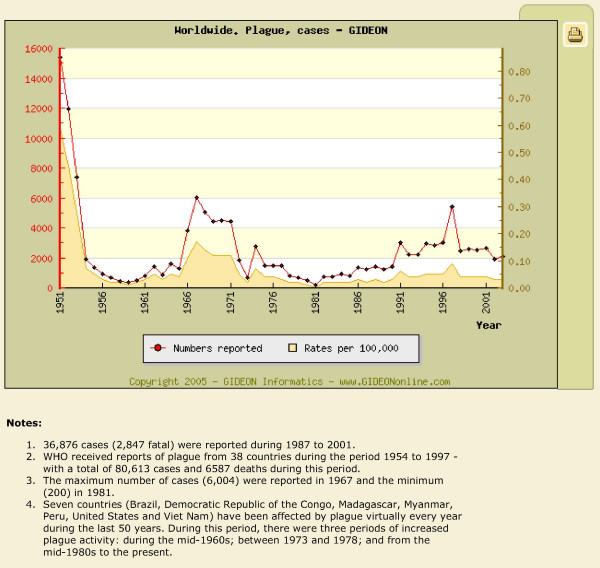
Plague – Worldwide incidence and rates per 100,000.

**Figure 9:**
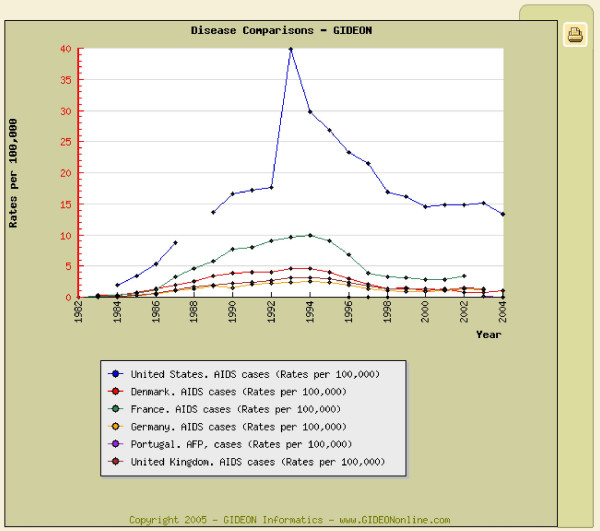
Graph contrasting AIDS rates among user-selected countries.

Maps which depict the global distribution of each disease can be accessed through the 'Distribution' tab (Figure 6). Text outlining country-specific data for the disease (Figure 7) is available through either a list of countries displayed in this module, or by clicking the relevant 'red dot' on the map. These text boxes also include data sets which automatically generate incidence / rate graphs (Figure 8), a chronological account of all disease outbreaks, and numbered reference links to relevant journal publications and reports of ongoing outbreaks from ProMed . A separate 'Graphs' option allows the user to generate custom-made graphs comparing multiple disease rates, or rates in multiple countries. (Figure 9).

**Figure 10:**
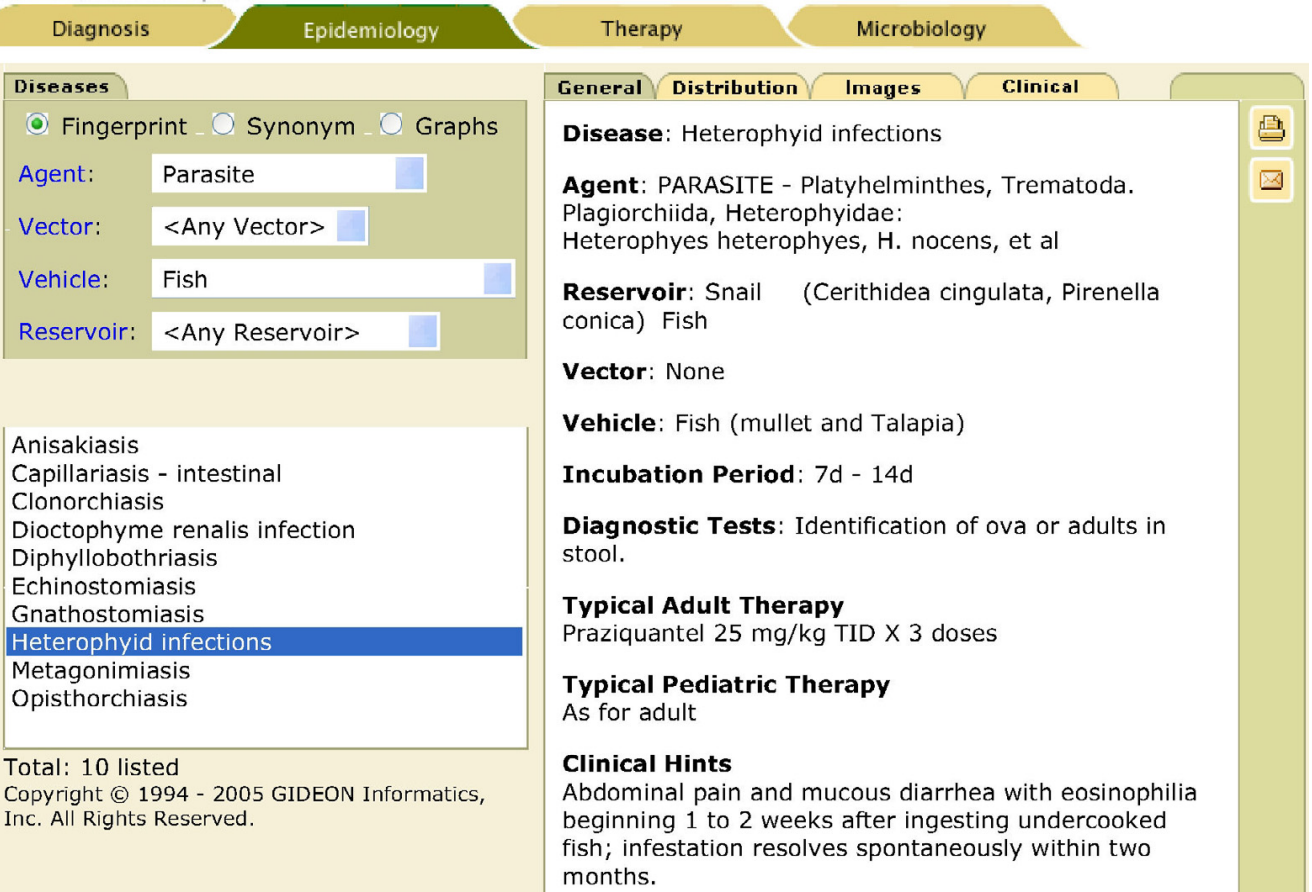
Epidemiology "Fingerprint" query (upper left screen). How many different parasitic diseases can be acquired by eating fish in Japan?

Additional tabs access the descriptive epidemiology and clinical background of each disease. Synonym tabs generate lists of alternative terms for diseases and countries in Spanish, German, Norwegian, etc. Historical data record the incidence of individual diseases and significant outbreaks spanning decades. An additional "Fingerprint" option generates a list of diseases compatible with any set of epidemiological parameters. For example, in Figure 10 we see that ten parasitic diseases are transmitted by fish in Japan.

## Therapy

The Therapy module follows the pharmacology and application of all drugs and vaccines used in Infectious Diseases. The current version contains 264 generic drugs and 64 vaccines. Various sub-modules present the mechanism of action; pharmacology, dosages, drug-drug interactions, contraindications, spectrum, and susceptibility testing standards. An international synonym lists contains over 9,500 trade names. As in other modules, users may add custom notes in their own language for each drug or vaccine: prices, resistance patterns, local trade names, etc.

## Microbiology

The Microbiology option is similar to the Diagnosis module. Users may enter any combination of phenotypic tests, and obtain a ranked probability list of compatible bacteria. The current version incorporates more than 1,300 taxa. The Microbiology module is also designed to list the phenotype, prior names, ecology and disease association for any organism, or compare the phenotypes of any combination of organisms selected by the user.

## Assessment

Since the graphic and mapping functions of GIDEON treat individual countries as whole units, data presentations lack a certain degree of "granularity." Thus, the differential diagnosis of fever in Venezuela will include malaria, even if the patient is living outside of the endemic, southern region. This problem is corrected to a large extent by text in the associated country-specific notes and the general knowledge base of the treating physician. In theory, the manufacturer could follow the incidence of each disease for every state, district, province and oblast; but variability would still exist according to occupation, rural vs. urban setting, season, etc.

An additional problem relates to the availability and quality of valid epidemiological data. Disease reporting varies widely from country to country. For example, AIDS reporting statistics from sub-Saharan Africa are generally inadequate. Where necessary, the spreadsheets used by GIDEON record published true estimates rather than questionable reports. In other instances, Health Ministry data conflict with reports of the World Health Organisation, a fact which is recorded in relevant GIDEON country notes. Occasionally, major diseases are not reported at all. For example, several recent cases of cholera in Japan originated from Thailand; but Thailand has not officially reported a single case in many years. Where possible, the GIDEON data base relies on published best estimates, and at times 'educated guesses' when data are entirely lacking. Thus, there are few published data for disease incidence in Togo, and the program is forced to rely on publications for neighboring Ghana.

The reader is referred to the GIDEON website  for an extensive listing of data sources, published reviews, technical background and pricing information.

## Competing interests

The author serves as a salaried Scientific Advisor to Gideon Informatics, Inc.
